# Life expectancy with and without cognitive impairment among Chilean older adults: results of the National Survey of Health (2003, 2009 and 2016)

**DOI:** 10.1186/s12877-019-1387-5

**Published:** 2019-12-26

**Authors:** Ximena Moreno, Lydia Lera, Francisco Moreno, Cecilia Albala

**Affiliations:** 1 Institute of Nutrition and Food Technology, University of Chile, Avenida El Líbano 5524, Macul, Santiago, Chile; 2 University of Santiago (USACH), Avenida Libertador Bernardo O’Higgins 1611, Santiago, Chile; 3 Environment Ministry, San Martín 73, Santiago, Chile

**Keywords:** Life expectancy, Health expectancy, Ageing, Self-rated health, Cognitive impairment

## Abstract

**Background:**

Chile has one of the highest life expectancies within Latin American. This is the first study to determine health expectancies in older populations in Chile, considering cognitive status as a health indicator.

**Methods:**

We estimated prevalence of cognitive decline among people aged 60 years and over based on the Mini-mental State Examination and the Pfeffer Functional Activities Questionnaire, with data from the National Survey of Health (2003, 2009, 2016). Life expectancy free of cognitive impairment was calculated using the Sullivan method.

**Results:**

At age 60, life expectancy free of cognitive impairment was more than 3 years longer for women, compared to men of the same age. Life expectancy free from cognitive impairment was higher for both men and women aged 60 in 2016 when compared to 2003 (2.1 and 2 years higher, respectively).

**Conclusions:**

Longer life expectancy in women was accompanied by more years free of cognitive impairment. Men expected to live a similar proportion of years free of cognitive impairment, compared to women. Common and standardised assessments of health status of older people should be adopted in Latin American studies, to allow for time-trend analyses and international comparisons.

## Background

Life expectancy (LE) at birth in Chile (80 years) is one of the highest of Latin America and the Caribbean [1]. Life expectancy at 60 years in Chile is the second highest (24.6 years) for women and the highest (21.0 years) for men in South America [2]. The demographic transition in Latin America has occurred at a more accelerated pace, compared to European and North American countries, with less time to adapt to the particular needs of an aging population [3]. Therefore, it is unclear if those years of life gained are accompanied by an extension of good health. Others have highlighted the need for epidemiological research to properly understand the aging process in Chile, considering the advanced demographic transition and fast economic, social and cultural changes [4]. Health expectancies can be used as an indicator of the proportion of years expected to be lived in good health, taking into account information on specific health indicators and mortality [5].

Cognitive impairment (CI), which is more frequent at older ages, affects not only the individual, but also family members and the health system, being associated with dependency, increased risk of adverse health outcomes, poorer quality of life and economic costs [6]. Insufficient awareness of the problem and the lack of common and appropriate diagnostic criteria, have resulted in the absence of accurate estimations of the prevalence of CI among the Latin American older population during the first decade of 2000 [7]. According to Fuentes & Albala [8], the prevalence of CI in 2009 among Chilean older people aged 60–64 years was 1.2%, and steadily increased with age, reaching 32.6% for those aged 85 years and above. The prevalence among people aged 60 and above was 7.0%, with higher rates among women (7.7%, men = 5.9%) and rural (10.3%, urban = 6.3%) populations. One study reported that in 2000, women at age 60 living in Santiago, Chile, could expect to live 20.1 years (total LE = 22.8 years) free of CI, and men of the same age had a LE free of CI of 18.3 years (total LE = 19.1 years) [9]. Andrade et al. [10] reported an increase in years of life expected to be lived free of CI, among men (14.8 to 17.1) and women (17.9 to 20.0) at 60 years in Sao Paulo, between 2000 and 2010.

The aim of this study was to estimate LE with and without CI among men and women aged 60 years and more in Chile, in 2003, 2009 and 2016.

## Methods

### Study design

The National Survey of Health (NSH) is a cross-sectional study with three waves of data collected in 2003, 2009 and 2016. In 2003, it included people aged 17 and above, and the second and third waves included those aged 15 and above. In 2003, the sample was stratified and randomly sub-sampled from participants of the Quality of Life and Health Survey [11]. The Quality of Life and Health Survey used household information collected in the National Census of 1992 as the sample frame and carried out a stratified cluster sampling [12]. The NSH 2003 used the same age-sex-region structure of the original sample frame, with the exception of one region that was oversampled to improve accuracy of estimations. In 2009 and 2016, stratified cluster sampling was employed, based on the master sample frame of the Chilean National Institute of Statistics and the Population and Housing Census of Chile [13, 14]. The samples for the three versions are nationally, rural and urban representative. Weights were calculated for the three samples, considering the complex study designs [12, 13, 15]. The overall response rate was 90% in 2003, 85% in 2009 and 67% in 2016 [12, 13, 16].

The protocol of each wave of the NSH was approved by the Ethics Committee of the Pontificia Universidad Católica de Chile (Pontifical Catholic University of Chile).

### Data collection and health indicators

A set of sociodemographic and health variables were collected through face to face household interviews. Cognitive status was assessed in the three waves with a shortened version (nine items) of the Mini-Mental State Examination (MMSE), with a maximum score of 19 and a cut-off point of 13 [17]. This instrument was derived from a previously validated version of the MMSE [18]. Quiroga et al. [19] showed that a combination of MMSE and the Pfeffer Functional Activities Questionnaire (PFAQ) had a higher sensitivity and specificity to detect CI among the Chilean older population. Hence, the criteria used to determine if participants had CI were MMSE< 13 and PFAQ≥6. When participants obtained a score of 12 or less on the MMSE, a relative or a person who lived in the household answered the PFAQ.

### Mortality data

Abridged life tables for the Chilean population were obtained from the Global Health Observatory Data Repository of the World Health Organization. Life tables for 2003, 2009 and 2016 were employed. The life tables had an age interval of 5 years, starting from 60 years.

### Statistical analysis

Considering the complex design of the studies, weighted sex-specific prevalences were estimated for each 5 year age group. Missing data for cognitive status (6.9% in 2003; 5.0% in 2016–17) were imputed using hot deck imputation, a non-parametric method that employs covariates to impute plausible values to missing observations [20].

This information was used to calculate years of life expected to be lived free of CI for each group, employing Sullivan’s method [21]. To calculate LE free of CI, we followed the proposal by Jagger et al. [22]. We considered the person years lived at 5 year age intervals, total life expectancy in specific age groups, and the total number of years lived with and without CI in that age group. The person years to be lived free of CI were calculated by multiplying the person years of life in a specific age group by the proportion of people without CI (1-weighted prevalence). The total number of years without CI was calculated by adding up the person years without CI of the successive age intervals. LE without CI was obtained by dividing the total years lived without CI by the number of people surviving to the beginning age of the respective age interval. The proportion of life expectancy free of CI, standard errors and 95% confidence intervals were estimated using the information described above. We carried out sensitivity analyses to test the robustness of our estimations. In these analyses, people with a score of 12 or less in the MMSE were classified as having CI.

Prevalences and logistic regression were estimated with Stata 15. LE and LE free of CI were calculated with R 3.6.1.

## Results

As observed in Table 1, a total of 1158 people participated in NSH 2003, 1390 in NSH 2010, and 2031 in NSH 2016. With the exception of men in 2003, at least 80% of participants in every year lived in the urban area. The level of education increased between waves, with more participants who had completed the first level of school (8 years). Women had lower education than men.

**Table 1:** Sociodemographic characteristics of the sample of the National Survey of Health, 2003, 2009 and 2016

	2003		2009		2016	
	Men	Women	Men	Women	Men	Women
	(*n* = 492)	(*n* = 666)	(*n* = 543)	(*n* = 847)	(*n* = 737)	(*n* = 1294)
Age groups (%)						
60–64	21.1	20.4	28.2	26.5	26.7	24.5
65–69	22.2	23.7	23.8	23.3	24.2	23.7
70–74	24.2	24.8	19.2	16.1	19.5	19.5
75–79	16.9	16.5	16.2	16.1	13.0	15.5
80–84	8.9	7.2	8.7	11.1	10.2	10.4
85+	6.7	7.4	4.1	7.1	6.4	6.6
Area (%)						
Urban	75.4	81.7	83.3	83.2	80.2	80.9
Years of education (%)						
<8	70.7	75.0	61.2	64.1	45.5	55.1
8+	29.3	25.0	38.8	35.9	54.5	44.9

Unweighted frequencies

Prevalence of CI was similar among men and women, and although point estimates decreased over time, no statistically significant decrease was observed between years, either for men or for women. As the decline in prevalence was statistically significant when data were not stratified (Table 2), this indicates insufficient statistical power to detect trends when stratified by gender.

**Table 2:** Prevalence of cognitive impairment among Chilean older men and women in 2003, 2009 and 2016

	2003		2009		2016	
	%	95% CI	%	95% CI	%	95% CI
Total	10.6	8.0–13.1	7.0	4.7–9.3	5.6	4.2–7.1
Men	10.8	6.9–14.7	7.8	3.3–12.2	5.8	3.8–7.7
60–64	4.1	0.5–7.8	2.5	0–5.1	0.8	0–2.0
65–69	4.4	0.9–7.8	3.9	0.5–7.3	2.7	0–7.0
70–74	12.4	4.0–20.7	6.2	0.2–12.2	4.7	0–14.3
75–79	20.0	4.4–35.6	12.1	1.2–23.1	8.9	1.7–16.1
80–84	25.3	4.2–46.3	22.4	0–48.2	17.5	4.9–30.1
85+	53.6	21.0–86.2	41.6	8.0–75.2	18.2	3.3–33.0
Women	10.4	7.0–13.8	6.4	4.2–8.5	5.5	3.5–7.6
60–64	1.1	0–2.6	0.4	0–1.3	0	0–0.3
65–69	2.7	0.3–5.1	1.7	0–4.1	0.1	0–2.0
70–74	10.4	2.3–18.6	3.3	0–8.3	2.4	0.2–4.7
75–79	12.5	4.8–20.1	5.4	0.9–9.8	14	3.5–24.4
80–84	30.4	4.1–56.7	26.6	13.7–39.5	14.2	6.3–22.0
85+	36.5	16.8–56.1	33.1	15.3–50.9	28.4	11.4–45.4

Weighted estimates

In logistic regression models adjusted by sex and age (see Table 3), people who had less than 8 years of education had a higher odds of CI in 2009 (OR = 3.8, 95% CI 1.6–9.4) and 2016 (OR = 2.9, 95% CI 1.5–5.5). Area of residence was not associated with CI.

**Table 3:** Odds ratio of cognitive impairment among Chilean older people in 2003, 2009 and 2016

	2003		2009		2016	
Variable (reference)	OR	95% CI	OR	95% CI	OR	95% CI
Sex (men)	0.7	(0.4.0–1.3)	0.5	(0.3–1.2)	0.7	(0.4–1.3)
Age	1.1	(1.1–1.2)	1.1	(1.1–1.2)	1.1	(1.1–1.2)
Education^a^ (8 or more years)	1.4	(0.8–2.6)	3.8	(1.6–9.4)	2.9	(1.1–5.5)

Weighted estimates

^a^Dichotomised as less than 8 and 8 or more years

**Fig. 1:**
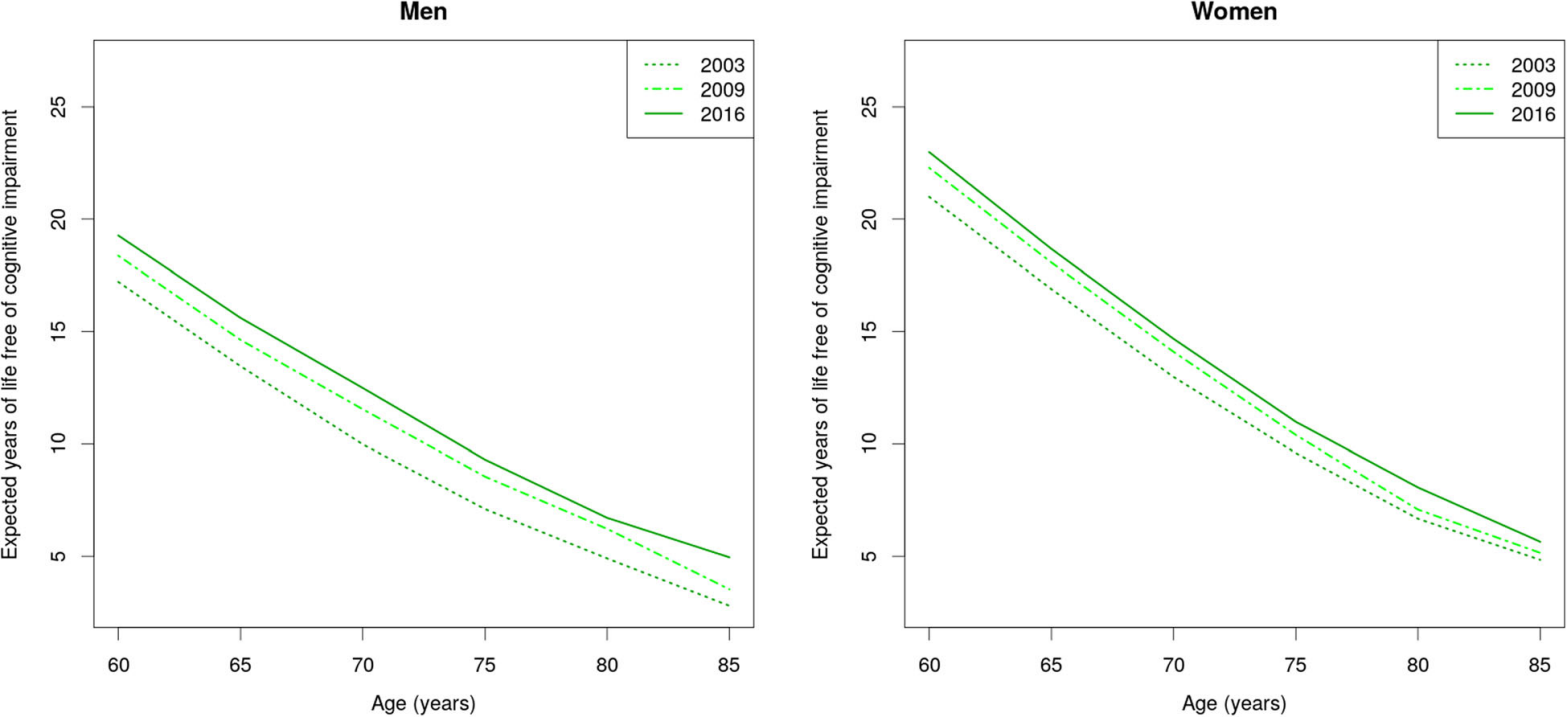
Life expectancy free of cognitive impairment among Chilean older men and women in 2003, 2009 and 2016

As shown in Table 4, LE was longer among women. Between 2003 and 2016, LE rose 0.8 years among women, and 1 year among men, reaching 25.3 years and 21.3 years, respectively. At age 60 women had almost 4 more years of LE free of CI than men. However, there was only a negligible difference in the proportion of years to be lived free of CI, between men and women. Figure 1 shows the sex difference in LE free of CI at different ages. In 2009, women aged 60 had a life expectancy free of CI (22.3, 95% CI 21.7–22.9) 2 years longer than men (18.4, 95% CI 17.8–19.0). At 75 years, a difference of almost 2 years was still observed (women = 10.4, CI 95% 9.7–11.1; men = 8.5, 95% CI 7.8–9.3). In 2016, women had higher LE free of CI (23.0, 95% CI 22.5–23.5) by more than 3 years compared to men (19.3, 95% CI 18.8–19.7). This difference decreased at older ages, but it persisted until the age of 80, with more than 1 year of additional LE free of CI among women (8.1, 95% CI 7.4–8.7) compared to men of the same age (6.7, 95% CI 6.1–7.3).

**Table 4:** Total life expectancy, life expectancy free of cognitive impairment and life expectancy with cognitive impairment at age 60 in 2003, 2009 and 2016 in Chile

	2003		2009		2016	
	Men	Women	Men	Women	Men	Women
Life expectancy (years)	20.3	24.5	20.5	24.9	21.3	25.3
HLE	17.2	21.0	18.4	22.3	19.3	23.0
(95% CI)	16.6–17.8	20.2–21.7	17.8–19.0	21.7–22.9	18.8–19.7	22.5–23.5
% HLE	84.8	85.7	89.6	89.5	90.5	90.9
(95% CI)	81.8–87-8	82.6–88.7	86.7–92.5	87.1–91.9	88.3–92.6	88.9–92.8
UHLE	3.1	3.5	2.1	2.6	2.0	2.3
(95% CI)	2.5–3.7	2.6–4.3	1.5–2.7	2.0–3.2	1.6–2.5	1.8–2.8
% ULE	15.2	14.3	10.4	10.5	9.5	9.1
(95% CI)	12.2–18.2	11.3–17.4	7.5–13.3	8.1–12.9	7.4–11.7	7.2–11.1

*HLE* life expectancy free of cognitive impairment, *UHLE* life expectancy with cognitive impairment

Changes in LE free of CI between 2003 and 2016 are also shown in Fig. 1. An increase of 2 years was observed among men (17.2, 95% CI 16.6–17.8 vs. 19.3, 95% CI 18.8–19.7) and women (21.0, 95% CI 20.3–21.7 vs. 23.0, 95% CI 22.5–23.5) at 60 years. This tendency was observed until the age of 70 for women (13.0, 95% CI 12.2–13.8 vs. 14.7, 95% CI 14.1–15.2), and to 85 years for men (2.8, 95% CI 1.8–3.8 vs. 5.0, 95% CI 4.3–5.6). Women aged 60 in 2016 had an increase of 5.2% points in the proportion of years to be lived free of CI compared with women aged 60 in 2003 (85.7, 95% CI 82.6–88.7% vs. 90.9, 95% CI 88.9–92.8%). An increase of 5.7% points in the same period was also observed among men of the same age (84.8, 95% CI 81.8–87-8 vs. 90.5, 95% CI 81.8–87-8).

The results of our sensitivity analyses showed that sex differences and time trends were similar to those observed in our primary analyses. As expected, prevalence of CI was higher and LE free of CI was lower for both sexes, when the MMSE alone was used to determine CI.

## Discussion

According to our results, women in Chile aged 60 in 2016 could expect to live 0.8 years longer than women who were aged 60 in 2003. Men aged 60 in 2016 could expect to live 1 year longer than their 2003 counterparts. Chilean women at age 60 expected to live more years without CI, compared to men across all waves of the survey. For both men and women, there was an increase in the years expected to be lived free of CI and in the proportion of years free of CI between 2003 and 2016–17.

The increase in LE between 2003 and 2016 in Chile was lower than in Brazil [10] and the United States [23]. Mathers et al. [24] have shown that mortality rates in old age in middle and high income countries, including Chile, have been consistently decreasing. Others have suggested that increases in LE at 50 years of age in Latin America are slowing, attributable to the impact of smoking [25]. As observed in other studies [9, 10, 26] we found that women at 60 years expected to live more years free of CI, compared to men. The greater LE free of CI among women, compared to men could be related to a later CI onset among older women [27]. The number of years expected to be lived free of CI was similar to what has been reported by other studies in Latin America [9, 10]. Studies from England [26] and the United States [23] report fewer years of LE free of CI at age 60 compared to our study. One explanation is that these studies included people aged 65 and over, at least 5 years older than the population in our study. Two studies from the United States [23, 28] found a higher prevalence of CI in older adults than we did. Our sensitivity analyses suggest that this is attributable to differences in the way that CI was defined. We used the scores of the MMSE alone to determine CI. With this measure, prevalence of CI increased and LE free of CI was lower for men and women. However, sex differences and time trends were similar to those observed in our primary analysis. The combination of MMSE and PFAQ scores which we used are a more accurate screening tool of CI for the Chilean older population than MMSE alone [19]. We found an increase in LE free of CI and a decrease in LE with CI between 2003 and 2016–17. It has been previously suggested that education level could have an impact on LE free of CI [10, 29]. Garcia et al. [30] found that more educated older adults, from different ethnic background, living in the United States were more likely to be free of CI. Nitrini et al. [31] observed that the prevalence of CI among illiterate older people in Latin America was twice as high compared to literate people. In line with this, we observed an improvement in educational attainment among Chilean older men and women during this period and the risk of CI was significantly higher among people with less years of education. The effect of education on LE free of CI among the Chilean older population warrants further examination. Some limitations of this study must be pointed out. In Chile, there are few longitudinal health surveys and the ones that exist are limited to specific regions of the country [32–34]. Only cross-sectional data were available to answer our research question. Our approach did not allow us to consider transitions between states of health or duration of each state of health. Instead, we based our calculations on prevalence at a specific point in time. Nevertheless, the Sullivan method is the most widely used method to estimate health expectancies, since population studies with longitudinal data are less common [35]. Second, institutionalized people are not included in the Chilean NSH, which might mean we have overestimated LE free of CI. In 2002, the estimation was 1.6% in the city of Santiago [36]. There are no updated estimations of the percentage of older people living in institutions. The first National Policy on Aging in Chile focused on extending family care, in order to delay entry into institutional settings [37]. The current National Plan of Dementia [38] emphasises family and community support. Hence, it is likely that the proportion of older people with CI who are institutionalised is low. Finally, the screening test for cognitive impairment was an adapted version of the MMSE in combination with the PFAQ. As discussed above, this makes international comparisons difficult. However, the cut-off point of the adapted version of the MMSE for the Chilean population has been validated [18], and the combination of the MMSE and the PFAQ results have been established as a screening tool for CI with good sensitivity and specificity [19]. Hence, our results provide an appropriate estimate of the years to be lived with CI among Chilean older people, to inform public health decisions. Our report also provides estimations of trends in LE with or without CI, which is an important strength of our study.

Unlike European countries and the United States, Latin American countries have recently started to estimate health expectancies among the older population. Our goal as a region should be to generate a similar set of knowledge to inform health policy decisions. This process calls for a joint effort, including researchers and public health authorities. It is necessary to identify existing data sources and to design population studies to gather information to estimate health expectancies on a regular basis, in order to complement the LE indicator with information about the health status of the population. It is important to determine and standardise specific measures of health status, to enable proper time-trend analyses and international comparisons.

## Conclusion

Longer LE in Chilean older women was accompanied by more years free of CI. Men and women expected to live a similar proportion of their remaining years of life free of CI. Latin American studies should adopt common and standardised assessments of health status of older people. This would make possible time-trend analyses and international comparisons.

## Data Availability

The datasets generated and/or analysed during the current study are available in the database repository of the Epidemiology Department of the Chilean Ministry of Health: http://epi.minsal.cl/bases-de-datos/ Life tables were obtained from the Global Health Observatory Data Repository of the World Health Organization, available from: http://apps.who.int/gho/data/view.main.LT62010?lang=en

## Abbreviations

CI: Cognitive impairment
LE: Life expectancy
MMSE: Mini-Mental State Examination
NSH: National Survey of Health
PFAQ: Pfeffer Functional Activities Questionnaire
